# Facilitating needs based cancer care for people with a chronic disease: Evaluation of an intervention using a multi-centre interrupted time series design

**DOI:** 10.1186/1472-684X-9-2

**Published:** 2010-01-11

**Authors:** Amy Waller, Afaf Girgis, Claire Johnson, Geoff Mitchell, Patsy Yates, Linda Kristjanson, Martin Tattersall, Christophe Lecathelinais, David Sibbritt, Brian Kelly, Emma Gorton, David Currow

**Affiliations:** 1 Centre for Health Research & Psycho-oncology, The Cancer Council NSW, University of Newcastle, Hunter Medical Research Institute & Priority Research Centre in Health Behaviour, Callaghan, Australia; 2 Discipline of General Practice, University of Queensland, Brisbane, Australia; 3 Institute of Biomedical Health Innovation and School of Public Health, Queensland University of Technology, Brisbane, Australia; 4 Research and Development, Curtin University of Technology, Bentley, Australia; 5 School of Medicine, University of Sydney, Sydney, Australia; 6 School of Medicine and Public Health, University of Newcastle, Callaghan, Australia; 7 Department of Palliative and Supportive Services, Flinders University, Adelaide, Australia

## Abstract

**Background:**

Palliative care should be provided according to the individual needs of the patient, caregiver and family, so that the type and level of care provided, as well as the setting in which it is delivered, are dependent on the complexity and severity of individual needs, rather than prognosis or diagnosis [1]. This paper presents a study designed to assess the feasibility and efficacy of an intervention to assist in the allocation of palliative care resources according to need, within the context of a population of people with advanced cancer.

**Methods/design:**

People with advanced cancer and their caregivers completed bi-monthly telephone interviews over a period of up to 18 months to assess unmet needs, anxiety and depression, quality of life, satisfaction with care and service utilisation. The intervention, introduced after at least two baseline phone interviews, involved a) training medical, nursing and allied health professionals at each recruitment site on the use of the *Palliative Care Needs Assessment Guidelines *and the Needs Assessment Tool: Progressive Disease - Cancer (NAT: PD-C); b) health professionals completing the NAT: PD-C with participating patients approximately monthly for the rest of the study period. Changes in outcomes will be compared pre-and post-intervention.

**Discussion:**

The study will determine whether the routine, systematic and regular use of the *Guidelines *and NAT: PD-C in a range of clinical settings is a feasible and effective strategy for facilitating the timely provision of needs based care.

**Trials registration:**

ISRCTN21699701

## Background

The delivery of appropriate and equitable care is a challenge facing many areas of health care, including palliative care. In fact, the health care experiences of people with life limiting illnesses have become a primary concern in recent years, with literature suggesting that the management of advanced cancer is lacking in terms of access [2]. Despite the increased attention, issues surrounding when and how palliative care should be delivered, as well as who should receive this care are yet to be resolved.

Palliative care has relied in part on prognosis or diagnosis based models to guide delivery of care. However, the need to provide palliative care in a more accessible and equitable manner has led to Palliative Care Australia (PCA) recommending a more needs-based model [1]. This model offers a way to improve the delivery of palliative care by triaging people with life limiting illnesses such as cancer according to complexity of their needs. Providing care on the basis of needs offers a way to ensure that the finite specialist palliative care resources available are provided to those people who need them most [3], while allowing less complex needs to continue to be met by primary care or allied health services. Hence, patients with minimal needs can continue to be cared for by their primary care team; while those with intermediate or complex needs may require the consultative or continued involvement of specialist providers [1].

However, implementing a needs based model has its own challenges, including how to define need and how and when to assess need [4]. Having best practice standards and pathways to referral may assist with identifying those who require the assistance of palliative care services and also those who no longer require specialist palliative care input [5]. The *Palliative Care Needs Assessment Guidelines*[6] (hereafter referred to as the *Guidelines*) were developed in an attempt to fill a gap nationally and internationally[6] by educating and informing health professionals about the issues that affect people with advanced cancer, their families and professional carers to facilitate timely referral to specialist palliative care services if required. As ensuring optimal compliance with guidelines is a primary concern for developers [7-11]; the *Needs Assessment Tool: Progressive Disease - Cancer (NAT: PD-C) *was developed to complement the *Guidelines*[6].

The NAT: PD-C was designed for ongoing use in both generalist and specialist care settings. Rather than simply determining who would benefit from a referral to a specialist palliative care service, the NAT: PD-C assists health professionals in matching the types and levels of need with the most appropriate person or service to address that need [12]. Psychometric properties of the NAT: PD-C were initially explored in a pilot study with simulated patients and caregivers [under its original name of the *Palliative Care Needs Assessment Tool*][12]. Further testing in a clinical palliative care setting confirmed the NAT: PD-C as a highly acceptable and efficient tool that can be used by health professionals with a range of clinical expertise [13]. However, the need for further evaluation of the *Guidelines *and NAT: PD-C to assess patients and their caregivers at multiple time points and determine the responsiveness of the NAT: PD-C to change over time has been acknowledged. In response to this, the research team undertook the following prospective, multi-site, multi-discipline longitudinal study.

The aims of the study were two-fold: to assess the impact of the systematic and ongoing use of the *Guidelines *and NAT: PD-C on patient and caregiver outcomes including level of need, quality of life, anxiety and depression and satisfaction with care; and to assess their impact on patient service use and referral patterns.

## Methods/design

### Ethical approval

The study was approved by the Human Research Ethics Committees of the University of Newcastle and of the Area Health Services of Hunter New England, Sydney South West, South Eastern Sydney and Illawarra.

### Study design

The study utilises an interrupted time series design [14], with data collected at multiple time points before and after the intervention is introduced [15]. By collecting data before and after the intervention, researchers can determine whether the intervention has an effect significantly greater than the underlying secular trend [15,16]. One advantage of this design is that it allows both the short-term and long-term effects of the intervention to be examined more akin to an effectiveness study than a more limited efficacy study [14]. Moreover, this design is highly suited for use in smaller populations and complex interventions [14].

### Participants

Inclusion criteria for patients are: (1) having a diagnosis of advanced cancer, ie, no longer amenable to cure, with either extensive local or regional spread, or metastatic disease; (2) being aged 18 years or older; (3) understanding English sufficiently well to complete questionnaires and telephone interviews; and (4) judged by the clinic staff as emotionally and cognitively capable of participating. Caregiver inclusion criteria are: (1) being nominated by the patient as the primary carer or family member who has provided, or may provide when needed, the most help to the patient; and (2) understanding English sufficiently well to complete questionnaires and telephone interviews.

## Sample size

The main outcome of interest in this study is each patient's level of unmet needs as measured by the Supportive Care Needs Survey - Short Form (SCNS-SF34) [17]. The percentage of people reporting at least one moderate or high need in each of the domains in the SCNS was calculated pre- and post-intervention. Having a moderate or high need was chosen as the category indicating a need as it has been suggested that "by evaluating psychosocial interventions with patients experiencing moderate to severe symptoms, future research is likely to yield findings of greater relevance to clinical practice" [18]. A systematic review of unmet supportive care needs in people with cancer reported that a number of studies have used a classification of moderate or high need to assess the prevalence of needs in people with cancer [19]. Many of these studies have reported prevalence for individual items (range 2-48%) rather than the prevalence of people with at least one need in each domain [20-23]. A recent study reported that the percentage of people with cancer identified as having at least one moderate or high need for help using the of the SCNS-SF34 ranged from 15% (sexuality domain) to 53% (psychological domain) [17]. Six spiritual items from the Needs Assessment for Advanced Cancer Patients (NA-ACP) [24]; a 132 item instrument assessing the needs of people with advanced incurable cancer [24], were assessed using the same response options as the SCNS.

Assuming a maximum prevalence of 50% (worst case scenario) at pre-intervention for all domains, it was calculated that using a 5% significance level and having a minimum of 407 patients would give the study 80% power to detect a reduction in prevalence of 10% in each of the five SCNS-SF34 domains and one NA-ACP domain post-intervention. However, this estimation of expected change in prevalence could not be supported by any previous literature, as few studies have looked at changes in needs over time or changes in the prevalence of needs resulting from an intervention [19].

Caregivers of all consenting patients will also be invited to participate in the study. Of an estimated 407 patients consenting to participate, it is anticipated that 60% will have a consenting caregiver (n = 245).

### Procedure

Participants will be recruited from the medical oncology, radiation oncology and haematology outpatient clinics from three major cancer centres in NSW, Australia. Initially, a research nurse will review clinic lists to identify patients who meet eligibility criterion (1). The list will then be reviewed by a clinician or clinic nurse to exclude patients who do not meet eligibility criteria (2), (3) and (4) and to make the initial approach to eligible patients. The research nurse will then provide a verbal and written explanation of the study to patients interested in the study and obtain their informed consent to participate. Consenting participants will be asked to nominate a caregiver and if the caregiver is present at the clinic at the time of recruitment, the research nurse will provide them with a verbal and written explanation of the study. If not, the caregiver information will be given to the patient who will be asked to pass it on to the caregiver. If the patient is unable to nominate a caregiver at the time of recruitment, s/he will be asked during the first and subsequent computer assisted telephone interviews (CATIs) to nominate a caregiver in case their circumstances change during the course of the study.

### Materials

Participants will complete bi-monthly CATIs over a period of 18 months. Primary outcomes include patient and caregiver unmet needs and patient service utilisation. Secondary outcomes include patient and caregiver anxiety, depression and quality of life and caregiver satisfaction with care.

#### Background questions

Socio-demographic questions will be asked including participant age, gender, marital status, level of education, type of health insurance, gross income and employment. Patients will also be asked about type of diagnosis and time since initial diagnosis; while caregivers will be asked about tasks performed in their care giving role as well as their relationship to and personal contact with the patient. A 12-item index [25] will assess co-morbid conditions, including cerebrovascular disease, inflammatory bowel disease, liver disease, gastric ulcers, arthritis, diabetes, depression, hypertension, chest pain, heart attack, heart failure, and chronic lung disease.

#### Primary outcomes

The 34-item Supportive Care Needs Survey - Short Form (SCNS-SF34) [17,26], plus six spirituality needs items and four additional needs items from the Needs Assessment for Advanced Cancer Patients (NA-ACP) [24]; will be used to assess the types and levels of unmet needs of patient participants. Patients will also be asked to report on their use of health care providers, support services and complementary and alternative medicines. For caregivers, the 44-item *Supportive Care Needs Survey -Partners and Caregivers *will be assessed (Girgis, Lambert & Lecathelinais: The Supportive Care Needs Survey for Partners and Caregivers of Cancer Survivors: Development and Psychometric Evaluation, submitted to *Psycho-Oncology*).

#### Secondary outcomes

Quality of life will be assessed using the two global questions from the European Organization for Research and Treatment of Cancer Quality of Life Questionnaire (EORTC QLQ-C30) instrument [27]; and anxiety and depression will be measured using the 14-item Hospital Anxiety and Depression Scale (HADS) [28]. A total HADS score will be obtained to quantify overall level of distress [29-31].

### Intervention

The intervention involves the systematic use of the NAT: PD-C to facilitate assessment and provision of needs-based care to address patient and caregiver physical and psychosocial concerns by health professionals involved in the care of people with advanced cancer. To support the uptake of the intervention, medical, nursing and allied health professionals at each of the recruitment sites will receive training in the use of the *Guidelines *and NAT: PD-C.

A systematic review of interventions to change provider behaviour found that guidelines were more effective if active educational interventions and patient-specific reminders were used to disseminate them [32]. The use of workshops and seminars were considered to assist in educating and training clinicians in the use of the *Guidelines *and NAT:PD-C, however research suggests that educational approaches using self-directed learning vary in effectiveness as health professionals vary in motivation to attend, change and self assess [33].

For this study, training in the intervention will be undertaken using an academic detailing approach, utilising individual as well as group sessions, as required. This approach involves a limited set of objectives delivered by expert trainers to individuals in their own environment at their own convenience to provide evidence based information regarding professional practices [34,35]. Group and individual academic detailing have both been shown to change health professional behaviours [36], particularly for those who received individual visits [37]. Academic detailing sessions are often more time efficient than workshops and seminars as they are brief, focused and delivered in the health professionals' own environment [38]; and health professionals have expressed a preference for this method along with audit and feedback [38].

#### Intervention resources

The *Guidelines *include reviews of the current referral practices and utilisation of palliative care services in Australia; patients' physical, psychological, social/cultural, spiritual and financial/legal issues; as well as caregivers/families' and health professionals' issues [6].

The NAT: PD-C aims to operationalise the *Guidelines *and includes four sections:

1. Section 1 includes three items to fast-track a review by a specialist palliative care service: the absence of a caregiver (if one is needed), a patient or caregiver request for referral to a specialist palliative care service, and the health professional's need for assistance in managing care;

2. Section 2 assesses the patient's wellbeing, and includes physical, daily living, psychological, information, spiritual/existential, cultural and social, financial and legal domains;

3. Section 3 assesses the ability of the caregiver/family to care for the patient, and includes physical, daily living, psychological, information, financial and legal and family and relationship domains;

4. Section 4 assesses the caregiver's own wellbeing, including physical, psychological and bereavement issues.

For Section 1, response options are "Yes" or "No". Items in Sections 2-4 are assessed according to the level of concern ("none", "some/potential for", "significant") they were causing. Prompt questions for each item were included on the back page, to facilitate consistency in how issues were addressed. Each item has a set of tick boxes to indicate the action taken ("directly managed", "managed by another care team member", "referral required") to address any identified needs. Finally, should a referral be required, the NAT: PD-C includes a section detailing the type of referral made (eg to specialist palliative care service, social worker, general practitioner, medical oncologist), the urgency of the referral ("urgent", "semi-urgent", "non-urgent") and client knowledge of the referral.

Once the training is completed at a particular site, the outpatient clinic appointment dates of each patient participant will be identified. Prior to each appointment, the research nurse will place a copy of the NAT: PD-C in the medical record of the patient; and will send an email to each clinician at the beginning of the week with a list of patients due to have NAT: PD-Cs completed at the next appointment. Health professionals at participating sites will be asked to complete NAT: PD-Cs in consultation with the patient, at every appointment for each patient participant, or approximately monthly if appointments are more frequent. Staff members of specialist palliative care services and allied health professionals will also be asked to complete the NAT: PD-C on each of the patients referred to them at initial assessment and, subsequently, monthly whilst they remain in contact with that patient. Finally, GPs will be sent a letter requesting that they complete the NAT: PD-C at the patient's next appointment, with remuneration offered to cover the extended consultation time.

### Analysis

Descriptive statistics of patient characteristics at baseline and summary measures of their level of need, anxiety, depression and quality of life at each time point will be presented as means and 95% confidence intervals for continuous variables and as proportions and 95% confidence where the data were categorical. The patients' baseline interview scores for each of these outcomes will be examined to determine whether scores varied according to age, gender, presence of a caregiver and level of care giving provided to them. Statistical significance will be assessed using chi square tests for categorical variables and t-tests for continuous outcomes (α = 0.05).

#### Changes in primary and secondary outcomes over time

For each patient, level of need, anxiety, depression and quality of life will be measured repeatedly during the study and therefore a Generalised Estimating Equation (GEE) model will be used to analyse the data [39]. In this study the GEE model will fit time as a factor and also analyse the number of CATIs completed as an interaction variable. Age, gender, time since diagnosis, co-morbidity score and presence of a caregiver will be included as potential confounding variables for patient participants. Age and gender will also be included as confounder for caregiver participants. For the purpose of this study, presence of a caregiver will include patients who had a participating caregiver at first assessment as well as patients who subsequently indicated they had a caregiver in any of their CATIs (irrespective of whether their caregiver consented to participate). GEEs will be run for both continuous and categorical outcome variables adjusting for the potential confounders listed above to ascertain whether the intervention had any impact on patient and caregiver outcomes.

#### Feasibility and acceptability of the *NAT*: PD-C

The uptake of the NAT: PD-C will be examined within the outpatient clinic setting by comparing the number of NAT: PD-Cs due for completion for each patient during the study (depending on the regularity of their appointments) with the number of NAT: PD-Cs that were actually completed. The impact of completing the NAT: PD-C on length of consultation in outpatient clinics will also be examined by audio-taping consultations in which the NAT: PD-C was completed and others in which it was not completed.

#### Service utilisation and referral patterns

The date of completion of each NAT: PD-C; the levels of need recorded on each NAT: PD-C; and the actions taken to meet identified unmet needs (including referrals to health professionals and/or services) will be identified. Using self-report CATI information, the mean number of health professionals to whom referrals were suggested at each time point pre- and post-intervention will be determined. Any change in the number of health professionals seen over time will be assessed using GEEs. Each patient's medical record will be audited to determine the dates of referral to health professional/services and dates on which the patient was seen by these health professional/services.

## Discussion

This study will assess whether the use of the Guidelines and NAT:PD-C will prompt a more comprehensive assessment of patient and caregiver concerns, potentially bringing about a reduction in the level of unmet needs, depression and anxiety; as well as an increase in patient quality of life. Moreover, it is hoped that these resources will prompt health professionals to address these unmet needs in a timely and appropriate manner either themselves or through referrals to other care team members or specialist providers. The study will examine the feasibility and efficacy of routine, systematic and regular use of the Guidelines and NAT: PD-C in a range of clinical settings in terms of their ability to facilitate the timely provision of needs based care for people with advanced cancer.

## Competing interests

The authors declare that they have no competing interests.

## Authors' contributions

AG and DC developed the study concept and aims and sought funding for the project. AW, AG and CJ developed the protocol and all of the other authors assisted in further development of the protocol. AW and AG were responsible for drafting the manuscript. AW, CJ and AG will implement the protocol and oversee collection of the data; and AW, CL, DS, AG and CJ will oversee/undertake data analyses. All authors contributed to the final manuscript.

## Pre-publication history

The pre-publication history for this paper can be accessed here:

http://www.biomedcentral.com/1472-684X/9/2/prepub
